# First description of extrafloral nectaries in *Opuntia robusta* (Cactaceae): Anatomy and ultrastructure

**DOI:** 10.1371/journal.pone.0200422

**Published:** 2018-07-17

**Authors:** Mario Alberto Sandoval-Molina, Hilda Araceli Zavaleta-Mancera, Héctor Javier León-Solano, Lupita Tzenyatze Solache-Ramos, Bartosz Jenner, Simón Morales-Rodríguez, Araceli Patrón-Soberano, Mariusz Krzysztof Janczur

**Affiliations:** 1 Research Group in Ecology and Evolutionary Biology, Department of Natural Sciences, Autonomous University of the State of Mexico, Toluca, Estado de México, México; 2 Instituto de Ecología, El Haya, Xalapa, Veracruz, México; 3 Unidad de Microscopía Electrónica, Postgrado en Botánica, Colegio de Postgraduados, Campus Montecillo, Montecillo, Texcoco, Estado de México; 4 Graduate Program in Agricultural Sciences and Natural Resources (PCARN), Autonomous University of the State of Mexico, Toluca, Estado de México, México; 5 Evidence Generation & Clinical Research RB, Hull, United Kingdom; 6 Instituto Potosino de Investigación Científica y Tecnológica, San Luis Potosí, México; Indiana University Bloomington, UNITED STATES

## Abstract

To our knowledge, there are no studies about the structure and ecological function of extrafloral nectaries (EFNs) in *Opuntia robusta*. This is the first description of EFNs in *O*. *robusta*, where young spines have an interesting structure and a secreting function, which are different from EFNs described in other Cactaceae species. We used light, scanning-electron, and transmission-electron microscopy to examine morphology, anatomy, and ultrastructure of the secretory spines in areoles in female and hermaphrodite individuals of *O*. *robusta*. Young cladodes develop areoles with modified and secretory spines as EFNs only active during the early growth phase. EFNs are non-vascularized structures, with no stomata, that consist of a basal meristematic tissue, a middle elongation region, and an apical secretory cone formed by large globular epidermal cells, containing nectar and medullar elongated cells. We observed the presence of Golgi apparatus, vesicles and plastids in the medullar and sup-epidermal cells of the spine. We propose that the nectar is stored in the globular cells at the apex of the spine and secreted by breaking through the globular cells or by pores. We recorded a more frequent presence of ants on younger cladode sprouts producing young secreting spines: this result is parallel with the predictions of Optimal Defense Hypothesis, which states that younger plant organs should be better defended than older ones because their loss produces a higher fitness impairment. Although Diaz-Castelazo’s hypothesis states that a more complex structure of EFNs correlates with their lower among-organs dispersion, comparing to less complex EFNs, non-vascularized structure of EFNs in *O*. *robusta* is not associated with their higher among-organs dispersion likened to *O*. *stricta*, which produces vascularized EFNs. We provide evidence that this characteristic is not a good taxonomic feature of *Opuntia* genus. Moreover, the comparison of EFNs of *O*. *robusta* and *O*. *stricta* suggests that the hypothesis of Diaz-Castelazo should be revised: it is rather a rule but not a law.

## Introduction

Many plant species produce extrafloral nectaries (EFNs) which are secretory structures present in different organs such as leaves, petioles, stipules, and young stems [1]. They are present either over the entire lifespan or only in young structures [2]. Contrary to floral nectaries, EFNs are situated outside the flowers and do not play a direct role in pollination, but they have a function in the maintenance of the mutualistic relationship between plants and arthropods. The most frequent relationship occurs between plants and ants in which ants are attracted to EFNs mainly by sugars, but also by small concentrations of amino acids and other organic compounds [3–7]. EFNs vary in their anatomy, composition, mode of nectar presentation, and ease of access for mutualistic arthropods [8–10].

The term EFNs is based on their ecological function rather than on their origin. Different types of EFNs may not be homologous: they share their glandular character [9] although they can differ in their structures; i.e. they can have a form of single-cell secretory hairs, complex cups, shallow bowl-like depressions or formless glandular tissue. Also, they can be highly vascularized or altogether lack vascularization [1].

In several species of Cactaceae, unique extrafloral nectaries were found in areoles; they appear to be modified spines and leaves [11] [12, 13]. In *Pachycereus schottii* (Engelm.) P.V.Heath, constitutive EFNs appear in fruits and buds, where the nectar was secreted from the tips of their tepals [14]. Almeida et al. [15] reported bristle-like bracteolar structures functioning as EFNs on stems of three subspecies of *Rhipsalis teres* Steud. Mauseth [16] observed, in *Ancistrocactus scheeri* (Salm-Dyck) Britton & Rose, EFNs formed from areolae meristem of modified spines. Apexes of tiny developing spines of several species of *Grusonia* (Reichenbach) produced a liquid collected by ants, but this secretion stopped once the spines grew larger [17]. In *Echinocactus polycephalus* Engelm. & J.M.Bigelow, EFNs were located above the areoles and, in *Carnegiea gigantea* (Engelm.) Britton & Rose, in the floral bracts [1]. In tubers of *Coryphantha clava* Lem., primordial leaves develop into glandular spines [11, 18]. The cactus *Carnegia gigantea* develops nectaries on buds, flowers, and fruits [19, 20]. *Tephrocactus alexanderi* (Britton & Rose) Backeb. and *T*. *articulatus* (Otto) Backeb. from Northwestern Argentina showed EFNs as modified spines in areoles of young vegetative and reproductive cladodes and on floral bracts [21]. EFNs visited by ants, were described on flower buds of two subspecies of *Neoraimondia arequipensis* (Meyen) Backeb. from Peru [22, 23].

One of the most studied cacti genera concerning the presence of EFNs is *Ferocactus* Britton & Rose. EFNs placed on the upper parts of the areoles were observed in *F*. *gracilis* H.E. Gates: their primary and secondary spines transformed in secretory tissue [24]. *F*. *acanthodes* subsp. *lecontei* (Engelm.) Lodé from central Arizona had permanent EFNs in form of glands subtending buds, flowers, or immature fruits [25]. *F*. *wislizeni* Britton & Rose in Arizona possesses secretory spines placed in a ring of areoles around the top of the cactus bearing flowers and fruits [20, 26–28].

EFNs were found in several species from the genera *Opuntia* MILL. and *Cylindropuntia* (Engelm.) F.M.Knuth. In *O*. *imbricata* Haw. EFNs were observed at the base of the spines of flower buds and fruits and on young vegetative stems [29]. Vegetative and reproductive buds of *O*. *acanthocarpa* Engelm. & J.M.Bigelow possess EFNs that are embedded in their areoles [30]. EFNs were also found in *O*. *engelmanii* Salm-Dyck ex Engelm. on areoles of new stems and buds, flowers, and fruits; in *Cylindropuntia leptocaulis* (DC.) F.M.Knuth on buds, flowers, and fruits; in *C*. *bigelovii* (Engelm.) F.M.Knuth on new stems only; in *C*. *fulgid*a (Engelm.) F.M.Knuth on areoles of old as well as new stems, and on areoles of buds, flowers, and fruits [19]; in *O*. *sulphurea* Gillies ex Salm-Dyck as modified spines on the areoles of new sterile cladodes and of cladodes supporting reproductive structures, as well as on the dorsal side of floral bracts [21]; in *O*. *stricta* (Haw.) Haw. in the areoles of the developing tissue of emerging cladodes, as well as in the areoles of developing flower buds [31–33]. *O*.*versicolor* Engelm. and *O*. *fulgida* also produce EFNs, but there is no accurate study of their structure [20]. Vascularized EFNs are present in *O*. *stricta*, but are unknown in others species from Opuntioideae subfamily.

There is controversy concerning the function of EFNs. Most of the researchers agree that they play a role in the indirect defense of either vegetative or reproductive structures [34]. However, the distraction hypothesis states that EFNs can also distract ants from attacking pollinators [35, 36]. Another hypothesis proposes that EFNs attract ant nests and thereby enhance plant nutrition [37]. For the defensive interaction between ants and plants producing EFNs to occur, ants must be present on the plant and show aggressive behavior towards potential herbivores, and there should be potential herbivore species. Additionally, the plant must be vulnerable to herbivore attack at least during some stage of its life. As the production of nectar requires energy, an optimal solution is an adjustment of the nectar flow to the herbivore activities [38]. Ants attracted by EFNs display an aggressive behavior towards herbivores [39] including large vertebrates [40] and, generally, more aggressive ant species provide a better protection against them [26, 41]. Moreover, the consumption of extrafloral nectar can enhance the aggressiveness of ants and their capacity to defend EFNs against potential competitors such as wasps and cheater ants [42] and change the foraging preferences of ants and thus their predation behavior [43, 44]. For some tropical ant species, extrafloral nectar is a most important source of sugar for the colony, and the proximity of plants producing nectar is critical for the colony and for maintenance of ants’ territory [38]. A particular case of this phenomenon is the close associations of territorial ants with the secretion of modified spines in *Ferocactus histrix* (DC.) G.E. Linds. 1955 and the construction of their nests on the top of the cactus [45].

Optimal Defense Hypothesis (ODH) states that plant resources are allocated in a way that optimizes the trade-off between costs and defensive benefits [46], that is, resources should be allocated to defense proportionally to the value of the plant tissue and the likelihood that a tissue will be attacked [47, 48]. Younger tissues are usually more valuable in terms of fitness than older tissues, because photosynthetic capacity of tissues declines with age [49, 50]. Some studies show that young leaves are better defended by chemical substances than older leaves [51]; however, older leaves may show better defense, mainly in species from tropical forests [52, 53], probably because young tissues require time to achieve higher defensive substance concentration [54], or young leaves can expand rapidly, shortening the period of greatest vulnerability of these leaves to herbivores at the expense of investment to defense [53, 55, 56] and can appear synchronously to satiate specialist herbivores [53, 57, 58].

Nectar secretion is affected by sunlight, soil moisture, and air humidity. Sunlight promotes photosynthesis and thus the synthesis of sugars involved in nectar production. Plants with sufficient water may invest part of it to nectar production [34], but lower relative air humidity promotes water evaporation from nectar and brings about a higher concentration of sugars, as compared to higher humidity [59, 60]. Nectars range from less than 10%, to more than 70% of sugar. The viscosity of nectar increases with higher sugar concentration. Higher viscosity may affect negatively the ability of visitors with sucking buccal apparatus to drink nectar; however, extrafloral nectar produced in sunny, dry environments may be viscous and does not affect visitors with open mandibles [34, 61]. The evaporation of the extrafloral nectar brings about a side effect: EFNs with more concentrated nectar are more attractive for ants when they are vulnerable to both higher air temperature and lower humidity. Since thermoregulation in ants is based on social interactions, nest type, size, material and placement, but not on the physiological response of an individual [62–64], it is difficult to imagine such mechanisms acting during their foraging on cacti sprouts. Therefore, ants should optimize their foraging strategy taking into account the fact that higher concentration of nectar compounds at higher temperature (lower humidity) allows for lower foraging time to obtain the same reward, but higher temperature also implies a thermoregulatory challenge.

During our previous study we observed the presence of ants on new cladodes and floral buds of *Opuntia robusta* H.L.Wendl., a naturally growing species in the municipality of Singuilucan, Hidalgo State, Central-Eastern Mexico [65]. *Opuntia robusta* is an endemic species from semiarid highlands of Meridional Plateau, Mexico. It is common in disturbed grassland on igneous soils, dominated by *Opuntia* “nopaleras” plants [45, 66]. The only reference about the presence of temporal EFNs in *O*. *robusta* was published by Zimmermann [2], but our study is the first one about morphology, anatomy, and cell structure of this type of nectary. *Opuntia robusta* is an interesting species, because it presents three sexual forms: masculine, feminine, and hermaphrodite, and at least three types of populations regarding sex compositions: strictly hermaphrodite, dioecious (masculine and feminine plants), and trioecious [67–69]. A possible gynodioecious population in Central-Eastern Mexico was suggested by Janczur et al. [65], but it must still be confirmed.

Provided that the anatomical and morphological structure of EFNs is considered an important feature for plant classification [70], its study is a longstanding topic in plant biology. Additionally, it has a further importance, since EFNs’ structure affects plants’ attractiveness to nectar-foraging insect visitors [33, 71] and thus also the outcome of the interactions among plants, their mutualistic defenders and herbivores. Diaz-Castelazo et al. [33, 72] state that more complex glands secrete more nectar, but are functionally homologous to the aggregations of numerous secretory trichomes on specific and valuable plant organs. Furthermore, more complex EFNs correlate with their lower among-organs dispersion, comparing to less complex EFNs. Higher among-organ dispersion is defined in their study as the presence of EFNs on different plant organs or/and the distribution of EFNs on the entire organ, i.e. trichomes on leaf surface. Following this concept, species from *Opuntia* genus have low among-organ dispersion, because EFNs are present generally on young cladode and flower sprouts, and occupy a determined zone, rather than the entire surface of an organ. They contrast simpler anatomy and morphology of among-organs dispersed EFNs, to more complex traits of EFNs circumscribed to determined organs. Furthermore, in the tropical vegetation of coastal dunes, there is another pattern: more complex morphology and anatomy of EFNs correlates with the presence of vascularization and, in turn, with their condition of being circumscribed. Only in two plant species, simple in structure (non-vascularized, unicellular) EFNs are circumscribed [72]. Plant species that have circumscribed glandular nectaries to particular organs produce larger nectar volumes per EFN than those plants with EFNs disperse among organs. EFN distribution among organs may affect ant visitation rate: circumscribed nectaries are more likely to attract a lower number of ant species than disperse nectaries [72]. If this is true, closely related species with different complexity of EFNs should reveal this rule.

The main goal of this study was to investigate the presence of extrafloral nectaries in young cladodes of *O*. *robusta*, and to explore their morphology and ultrastructure using light and electron microscopy in order to propose a mechanism of nectar secretion. Also, we tested whether ants prefer smaller and younger cladode sprouts with immature spines, as predicted by ODH.

## Materials and methods

### Ethic statement

This research did not involve measurements on humans or animals. We obtained the permission of the head of the Municipality of Singuilucan, State of Hidalgo, Mexico (Secretario General Municipal de Singuilucan, Estado de Hidalgo, México) to carry out research activities on the lands administered by the Municipality. The owners of the land gave us permission to conduct the study on this site and were informed about the permission from the Municipality. We obtained the statement from the Ministry of Environment and Natural Resources of the United States of México (Secretaría de Medio Ambiente y Recursos Naturales) which stated that no permission is necessary for plants from *Opuntia* genus. The study site is not considered a protected area [73], and *O*. *robusta* is not considered an endangered species [74, 75]. During the study, we did not affect or involve any endangered species. No plant was killed or severely damaged as a result of our research activity; the plant material used for this study was sampled only at a very limited scale; therefore, sampling had negligible effects on broader ecosystem functioning.

### Study species

*Opuntia robusta* is a plant form Cactaceae family. A most conspicuous characteristic of the plants from this genus is the presence of cladodia (cladodes) that are photosynthetic flattened branches or portion of a stem that functions as or resembles a leaf. Cladodes produce on their surfaces and borders groups of areoles that are small bumps out of which grow clusters of spines, which are presumably defenses against vertebrate herbivores. This species is almost endemic to Mexico, however it is widely distributed in arid and semi-arid regions of the country, in xerophyllous and crassulacean vegetation (cactus-dominated scrubland) [76], but also in *Pinus* and *Quercus* forests and grasslands of the states of Aguascalientes, Chihuahua, Coahuila, Durango, Guanajuato, Hidalgo, Jalisco, México, Michoacán, Morelos, Nuevo León, Puebla, Querétaro, San Luis Potosí, Sonora, Tamaulipas, Tlaxcala, Veracruz, Zacatecas, and México City. Also, it is found in Arizona, US [75, 77, 78]. Hernández et al. [75] stated that the number of populations of this species was increasing in the Singuilucan Municipality, but most of its habitat was already fragmented by croplands. Future disappearance of the population from San Nicolas Tecoaco, Singuilucan Municipality, is possible. Many plants studied by Janczur et al. [65] were removed to extend the farmland. At the study site the blossoming of *O*. *robusta* begins in late February and ends in late July. In our previous study [65], we did not find differences either in architecture or in size of the last-order (apical) cladodes between hermaphrodites and females. However further studies [79] showed that female cladodes were on average 12 and 10 cm shorter, for first and fifth order (basal and apical), respectively, than hermaphrodite cladodes. Hermaphrodite cladodes from different orders counting from soil differed in length: first through fifth order cladodes reached 30–47 cm, 30–45 cm, 25.5–39 cm, 25–40 cm, and 25–37.5, respectively, being the first order (basal) cladodes the oldest ones, and the closest to the soil.

Most of the pollinators of *O*. *robusta* are bees from Anthophoridae, Megachilidae, Halictidae, Apidae and Andrenidae families [67, 80].

The classification of *Opuntia* genus is rather complex. The resolution of molecular techniques is not high enough to find genetic differences between different *Opuntia* species. For example, Samah et al. [81], using 88 accessions and 13 SSR markers, found significant genetic differences between *O*. *robusta* and two groups: one of them constituted by *O*. *ficus-indica* (L.) Mill., 1768, *O*. *albicarpa* Scheinvar, *O*. *megacantha* Salm-Dyck, *O*. *streptacantha* Lem., *O*. *lasiacantha* Pfeiff., and *O*. *hyptiacantha* F.A.C. Weber, and the other one constituted by *O*. *joconostle* Web. and *O*. *matudae* Scheinvar, which, in turn, was different from others species.

We studied the EFNs from areoles of the youngest cladodes of hermaphrodite and female individuals of *O*. *robusta*, developed at the distal part of a branch, referred to as last-order cladodes. We investigated morphology, anatomy, and ultrastructure.

### Study site

We carried out the study in a slightly disturbed crasicaule scrubland with predominant *Opuntia* spp. (*O*. *robusta*, *O*. *sptreptacantha*, *O*. *spinulifera* Salm-Dyck, *O*. *megacantha*, *O*. *ficus-indica*, near village), *Ferocactus latispinus* Britton & Rose, *Mammillaria* sp. Haw., *Juniperus* sp. L., and *Agave* sp. L., in San Nicolas Tecoaco, Municipality of Singuilucan (20 2’ 38.2”N, 98 35’ 16”W) located at Pachuca mountain range, State of Hidalgo, at 2600 masl We performed the field work between February and June 2015.

### Ants’ activity

We chose 31 hermaphrodite plants with cladode sprouts one day before observation of ant activity on sprouts, using some of the 104 plants marked during our previous study [65]. We used only hermaphrodite plants, because females are very rare at the study site. Additionally, during this study we did not know the sex of some individuals determined in following seasons as females, since they did not blossom in 2015. Some of the females sampled in our previous study [65] were destroyed in following years, because part of their habitat was converted to farmland. So, we used female juvenile cladodes only to study the anatomy and morphology of their EFNs. Currently, we know 15 females in a population of several hundreds of individuals. We tagged each plant with a permanent marker, on a surface of chosen cladodes and the codes of the cladode sprouts, on the surface of their parental cladode, below each sprout. On June 15, 2015, we randomly chose one sprout per plant when a plant produced more than one sprout; we measured the length, width, and thickness of each sprout, using a caliper. During the next day of field work, over 1 min, we observed the number of ants that visited the sprout, once for each plant. Afterwards we moved to the next plant. We recorded air temperature and relative air humidity using a digital thermohygrometer, and the time when the observation started. The first observations began at 08:43, and the last observation finished at 15:44.

To test whether the number of ants on sprouts depended on sprout size, air temperature, air humidity, and time of day, we used Poisson Multiple Regression Model (PMRM): the response variables were modeled using maximum likelihood criteria. We assumed that the probability *P* to find ants on sprouts followed the Poisson distribution. We tested the effect of each predictor (sprout length, width, and thickness, air temperature and humidity, sampling time) in Poisson multiple regression, by removing it from the model and by comparing the adjustment of the model after and before the removal of this predictor (Type III test of fixed effect). The negative sign of a linear estimate in the PMRM means that the number of ants per sprout decreases with the inclusion of the corresponding effect to the model, provided that other variables are maintained constant. We corrected statistical tests of effects for slight over-dispersion seen in the data. We used *t* test for the significance of the model parameters [82, 83]. The PMRM results were generated using SAS software (Procedure GLIMMIX) [84]. To illustrate the relationships between pairs of variables and to estimate the probability of the linear, quadratic, and cubic effects, we used least square regressions [85]. To obtain the least square equations as well as the coefficients of determination (R^2^) for the relationships between ant number, or air humidity, or temperature and sampling time, we transformed the variable “time” as follows: *h*´ = *h* × 24—min(*h*) × 24, where *h*´ –transformed time, *h*–time in original units, min(*h*)–the earliest sampling hour. In all statistical tests, we assumed the significance level *P* = 0.05: we used the term “at the edge of significance” or “close to be significant”, when the probability to obtain a given result was slightly higher than *P* = 0.05, but less than *P* = 0.1.

To test whether ants got aggressive in the presence of invasion, we touched a chosen cladode sprout from each plant with grass stalk and observed ants’ reaction. Additionally, we observed ant behavior in the presence of herbivores on either cladode sprouts or cladodes parental to these sprouts.

To determine ant species that visited EFNs, using entomological tweezers, we sampled 2–5 individuals, from cladode sprouts, placed on different branches than those used for the ants’ exclusion experiment described in the next section. We placed the sampled individuals in 1.5 ml tagged Eppendorf tubes with 70% ethanol. In the laboratory, we determined their genus and/or species [86, 87].

### Extrafloral nectar sampling

In the attempt to obtain extrafloral nectar, we randomly chose 8 hermaphrodites and three feminine plants with cladode sprouts (different plants than those used for ants’ activity observation). To exclude the nectar-feeding arthropods, between 17:00 and 18:00, we applied tanglefoot around each sprout, on the surface of the parental cladode. At approximately 18:00 we covered it with nylon bags, to allow for the accumulation of the nectar. After 14 h, we uncovered the sprouts and used 2 μL capillaries to suction the accumulated nectar. If the nectar was too viscous to permit measurements, we applied a known amount of destilled water to the nectaries, collected the solution with capillaries, and calculated the sugar concentration before dilution (*C*_1_) using the following formula: *C*_1_ = (*V*_2_*C*_2_)/*V*_1_, where *V*_1_ and *V*_2_ –volume [μL] of distilled water before and after dillution, respectively, C_2_ –sugar concentration in the diluted solution [33].

### Plant and cladode sampling

For microscopic analyses we randomly chose one cladode sprout from each of three hermaphrodite and three female individuals. We took tissue samples from three areoles of each sprout. We determined their sex according with del Castillo & González-Espinosa [69] during 2010 and 2014. We used only plants with young distal cladodes (smaller than 12 cm long) and transported young cladodes, from the field to the laboratory, in a cooling (4 °C) container.

### Plant and ant photography in field

We took videos of ants on young *O*. *robusta* cladodes between 10:00 and 12:00 using a Nikon D3200 camera mounted on tripod, with AF-S DX NIKKOR 18-55mm f/3.5–5.6G VR lens (Nikon Inc.) mounted on a reverse ring.

### Light microscopy

We used young cladodes (smaller than 12 cm long) of parental plants. We removed three areoles from one young cladode of each of three plants (n = 9) using a razor blade and fixed them with FAA solution (3.7% formaldehyde, 48% ethanol, 5% acetic acid, in deionized water) for 72 h according to Ruzin [88]. We washed the tissues three times with water and preserved them in GAA solution (25% v/v glycerol, 50% v/v ethanol, 25% v/v water). For morphological description, we observed and characterized areoles using a Nikon SMZ800 stereomicroscope (Nikon Instruments Inc., Japan) and documented images with a Moticam 5MP (Motic Asia, Hong Kong). For anatomy, we sectioned the areoles, prepared them for transversal sections and processed them for standard paraffin embedding protocol using Paraplast Plus (McCormick Scientific, St. Louis, MO, USA) following Ruzin [88]. We obtained transversal (8 μm) and serial sections with a rotary microtome (American Optical Company, USA) and stained them with safranin O solution (0.05% safranin O C.I. 50240 in 3% NaCl in water) and fast green FCF solution (0.12% fast green FCF C.I. 42053 in 95% ethanol) following Zavaleta-Mancera et al. [89]. We observed the sections with a Axiostar Plus light microscope (Carl Zeiss, Germany) and captured images with a Moticam 5MP camera (Motic Asia, Hong Kong). We documented the procedure digitally [90].

### Scanning Electron Microscopy (SEM)

We removed four areoles from one young cladode (smaller than 12 cm long) of each of three plants (n = 12) using a razor blade. Each areola was cross-sectioned and fixed in glutaraldehyde solution (2.5% glutaraldehyde in 0.1 M phosphate buffer Sorensen's at pH = 7.2) for two hours under vacuum. We post-fixed the tissues in 1% osmium tetroxide in water at 22 °C for two hours. After two washes (1 h each) with deionized water, tissues were dehydrated in an ethanol series and critical point dried using a Samdri-780^®^ (TOUSIMIS Research Corporation, Rockville, USA). We coated the samples with gold:palladium (80%:20%) in a JFC-1100 (Fine coat ion sputter JFC-1100, JEOL Ltd., Tokyo, Japan) and observed them with a SEM microscope (JSM 6390 JEOL, Japan) working at 15 kv [91]. We documented the procedure digitally [92].

### Transmission Electron Microscopy (TEM)

We removed one areola from one young cladode (smaller than 12 cm long) of each of three plants (n = 3) using a razor blade. We removed fragments of 2–3 mm^3^ from each areola containing the secretory spines and fixed them in glutaraldehyde solution (2.5% glutaraldehyde in 0.1 M phosphate buffer Sorensen's at pH7.2) for two hours under vacuum. We post-fixed the fragments in 1% osmium tetroxide in water at 22 °C for two hours. Afterwards, we washed the samples twice with phosphate buffer and dehydrated them in an ethanol series (30%, 40%, 50%, 60%, 70%, 80%, 90% and 100%). We embedded the dehydrated tissues in medium hardness Spurr's resin (Polysciences Inc., PA, USA) according with manufacturer.

Semi thin sections (1.5 μm) were obtained with glass knife and an ultramicrotome (Ultracut, Reichert-Jung) and stained with a mixture 1:1 (1% methylene blue in 1% borax: 1% azure II in water). Thin sections (80 nm) were stained with 2% uranyl acetate in ethanol and Reynold’s Lead Citrate Solution according with Zavaleta-Mancera et al. [93]. We conducted microscopic analysis under a TEM microscope (JEOL 200 CX, Jeol, Japan) at 80 Kev at the Instituto Potosino de Investigación Científica y Tecnológica (IPICYT), and (JEOL JEM 12000 EII) at 80 Kv at the Instituto de Fisiología Celular, Universidad Nacional Autónoma de México (UNAM), and documented digitally [94].

### Fluorescent staining for callose

Callose (β-1→3-glucan) is the distinguished polysaccharide present on the sieve plates of the elements of the phloem tissue [95, 96]. Aniline blue stains specifically the callose and is used as a marker for identification of sieve elements of the phloem. We stained semi thin sections (1 μm) from the base of the areole with 0.5% of aniline blue in Sørensen’s phosphate buffer (0.1 M, pH = 8.0), for 1 h, under low light intensities (~5 μmol photons m^-2^ s^-1^), to prevent degradation of the dye. We observed the tissue under an Epifluorescence Microscope (Axioscope 2 Plus, Carl Zeiss AG) with a Set 01 (excitation: band pass (BP) 365/12 nm; emission: long pass (LP) 397 nm), connected to an Axiocam MRc5 camera. Callose of sieve elements stains bright green-blue [97].

## Results

### Ants’ activity

We observed ants’ activity on cladode sprouts: they searched mainly the apical secretory cones of young spines in areoles. We observed the secretion of transparent liquid, which attracted and fed ants (S1 Video). We identified the following ant genera and species: *Forelius* Emery, 1888 sp., *Camponotus* Mayr, 1861 sp1., *Camponotus* sp2., and *Camponotus* atriceps (Smith, F., 1858). Ants on sprouts were present in a broad range of temperatures (16–34.8 °C) and relative humidities (27–86%; S1 Table) [98].

**Fig 1:**
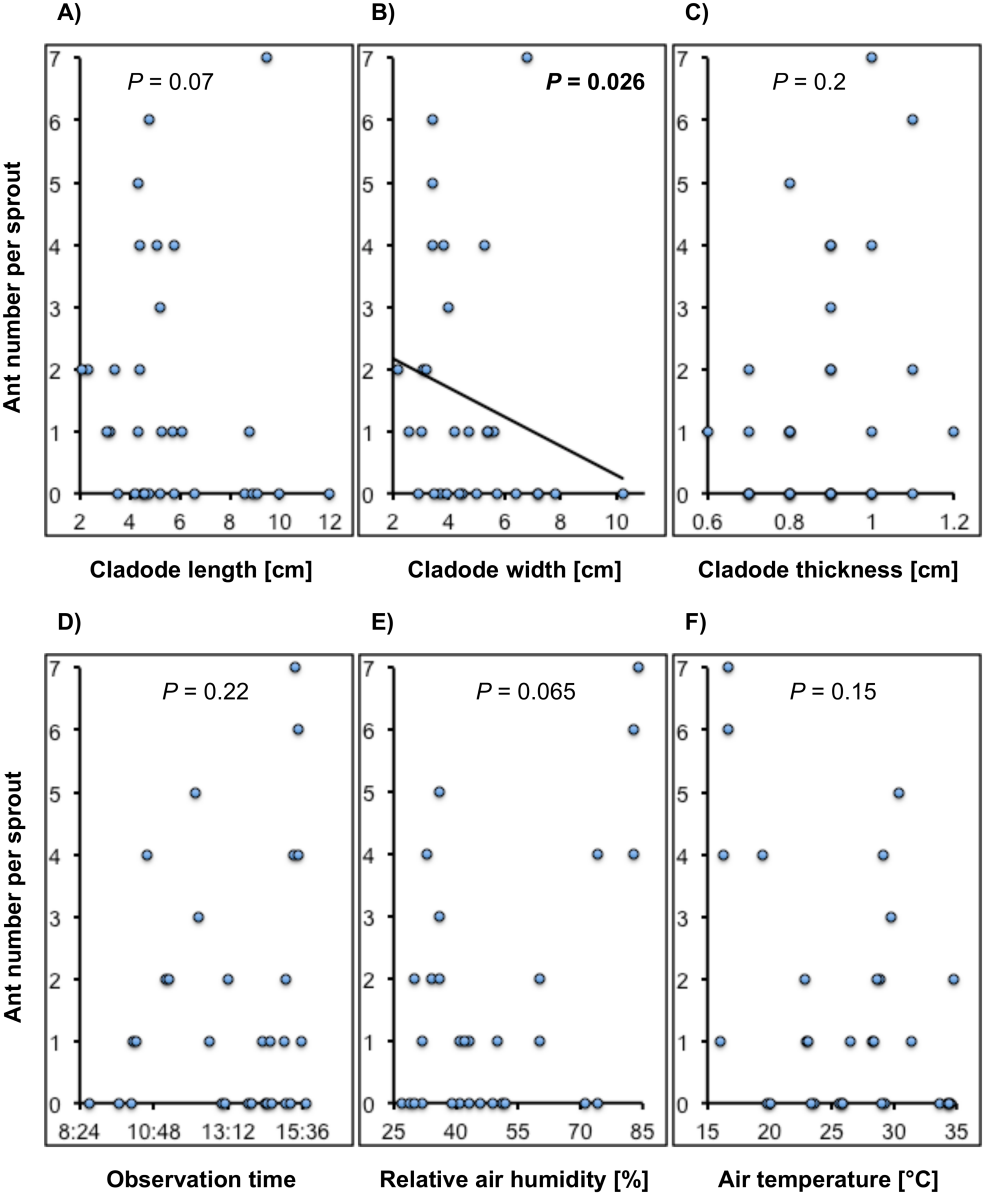
The effect of (A)–(C) sprout size (length, width, thickness), (D) time of observation, (E) relative humidity, and (F) temperature, on per sprout average ants’ number. Ants were less frequent on wider sprouts (B), and more frequent in the afternoon, when humidity was higher ((E); at the edge of significance: *P* = 0.065). We present the probabilities obtained in Poisson fixed-effect multiple regression model (PMRM). Regression equation for the significant relationship is depicted with continuous line, and its respective probability of contribution to the PMRM, with bold.

Since we determined three ant species only to genus level, we were not sure whether one or more species foraged on cladode sprout simultaneously. As far as we could distinguish ant species with the naked eye, we believe that only one species foraged on each cladode sprout during observation time. We did not observe ants on sprouts wider than 7 cm (S1 Table) [98]. The data from plants 40c-B and 89 were outliers (observations carried out in early morning), so were removed from the PMRM analysis [99]. Type III test of fixed model in PMRM showed that the inclusion of the cladode width was negatively associated with the number of ants on sprouts (Table 1), that is, ants visited narrower cladodes more frequently. The inclusion of either sprout length or sprout thickness with other variables being constant, did not improve the adjustment of the model, even when the simple regression between ant number and sprout thickness explained a larger percentage of variability than the analogous regression for sprout width. Notice that the inclusion of this variable to the Poisson regression improved the adjustment significantly, even when the model of the simple quadratic regression explained only 5% of the variability (Table 1; S1 Table; Fig 1A–1C) [98]. The use of the linear estimates for cladode size estimators in the PMRM model allowed for easier interpretation of the direction of their effects than the use of non-linear estimates. Additionally, it was justified because the change from linear to non-linear model in the simple regression modified only slightly the coefficients of determination: from 1.5% to 1.8%, from 4.8% to 4.9%, as well as from 7.7% to 8.4%, for linear and non-linear models, for sprout length, width, and thickness, respectively (S1 Table) [98].

**Table 1:** The effect of sprout length, width, and thickness, air humidity and temperature; on ant number on sprout, during 1 min observation.

Effect	Cladode size [cm]	Relative air humidity [%]	Air temperature [°C]	Estimate	df	t	*P* > |t|
Intercept				-33.67	20	-1.14	0.267
Size	Length			0.52	20	1.9	0.072
Size	Width			-0.96	20	-2.41	**0.026**
Size	Thickness			2.17	20	1.32	0.202
Humidity		Linear		-0.44	20	-1.8	0.087
Humidity		Square		6.0 x 10^−3^	20	1.95	0.065
Temperature			Linear	3.07	20	1.47	0.157
Temperature			Square	-5.0 x 10^−2^	20	-1.51	0.147
Time				-8.0 x 10^−5^	20	-1.26	0.222

We established that the probability to find ants on sprouts followed the Poisson distribution (Poisson regression). The effect of sprout width was negative and significant (bold). Ants were less frequent on wider sprouts. For cladode size estimators we adjusted the linear model. For relative air humidity and temperature, we tested both linear and square effects. The effect of both linear and quadratic components of humidity on the number of ants was on the edge of significance (*P* = 0.087 and *P* = 0.065, for linear and quadratic effect, respectively).

The estimate calculated from the inclusion of the quadratic effect of humidity to the model had an effect on the number of ants only at the edge of significance (*P* = 0.065), when other variables were controlled, even when the simple quadratic regression between these two variables explained 29% of the variability (Table 1; S1 Table) [98]. The inclusion of the temperature to the Poisson regression did not improve its adjustment, even when simple quadratic regression model explained 97% of the relationship between air temperature and relative air humidity (S1 Fig; Table 1; S1 Table) [98]. Quadratic and cubic regression models explained 82% and 89% of the relationship between air humidity or air temperature, and sampling time, respectively (S1 Fig; S1 Table) [98]. Air temperature was not associated with the cladode length and width (R^2^ = 1%, *P* = 0.6; R^2^ = 0.2%, *P* = 0.8, respectively; S2A–S2C Fig; S1 Table) [98]. Relative air humidity was not associated with the cladode length and width (R^2^ = 1.3%, *P* = 0.5; R^2^ = 0.7%, *P* = 0.7, respectively; S2D and S2E Fig). The increase of cladode thickness with the increase of either air temperature or relative air humidity, was explained by 13% (*P* = 0.05,) or 14% (*P* = 0.04) of these relationships, for temperature and humidity, respectively (S2C and S2F Fig; S1 Table) [98]. A linear relationship between cladode sprout length and width explained 88% of the variability (*y* = 0.72*x* + 0.59, *P* < 0.0001; S1 Table) [98].

There were more ants per sprout in the afternoon, when humidity was higher and temperature was lower, however the dispersion of the data was considerable and the effects of time, humidity and temperature were non-linear (Table 1; Fig 1D–1F; S1 Table) [98]. We had not observed ants on cladodes that did not produce new cladodes. Also, we observed ants on few plants with non-apical new cladode sprouts.

When we touched a cladode sprout with a grass stalk, ants got aggressive and in all the attempts they attacked the stalk. However, we have not observed ants’ attacks on true herbivores, such as *Chelinidea* sp. Uhler 1863 bugs (Coreidae) [100], or Coleoptera such as *Cactophagus spinolae* (Gyllenhal) (Dryophthoridae) [101], or *Euphoria basalis* Gory and Percheron, 1833 (Cetonidae) [102, 103], even when ants stayed close to them.

### Extrafloral nectar sampling

We could not obtain non-dissolved extrafloral nectar with capillaries, since it was too viscous to be suctioned. Neither could we dissolve it with distilled water, because the amount of nectar in the EFN was scarce.

### Morphology and anatomy

**Fig 2:**
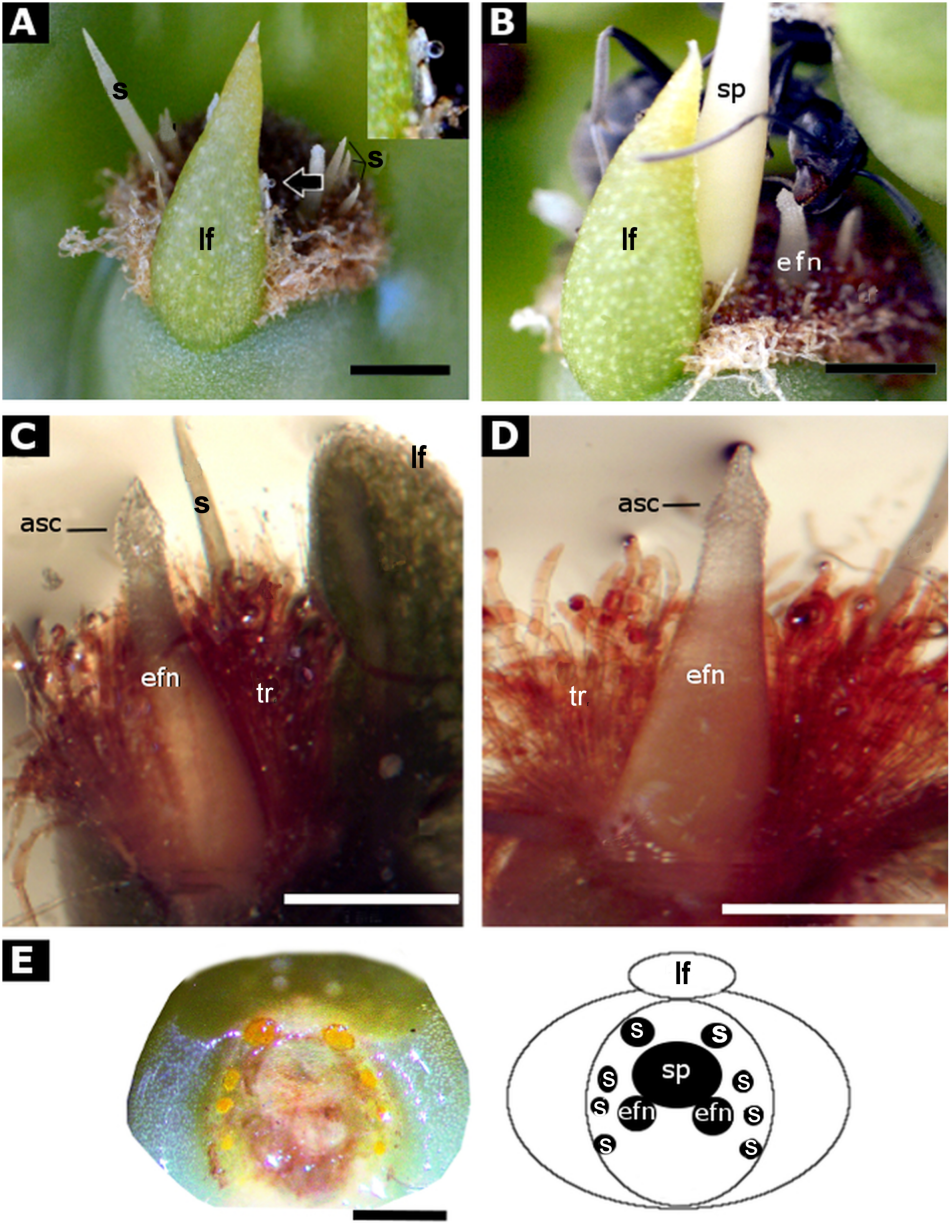
Areole of young cladode of Opuntia robusta. (A) Young areola with extrafloral nectary (efn, arrow) behind a persistent leaf, showing a drop of nectar (close up at the corner); (B) Ant collecting nectar from the extrafloral nectary; (C) and (D) Extrafloral nectary is a modified spine with an apical secretory cone (asc); (E) Distribution of the areola components and diagram. asc–apical secretory cone; efn: extrafloral nectary; lf—leaf; s—lateral spines; sp—central spine; tr—trichomes. (A) and (B)–bar = 2 mm, (C) and (D)–bar = 1mm, (E)–bar = 3 mm.

Extrafloral nectaries of *O*. *robusta* are modified young spines, placed in distal areoles of young cladodes of hermaphrodite and female plants (Fig 2A and 2B). We found two modified and secreting spines (1.76 ± 0.41 mm long) in each areola behind a persistent leaf and a larger spine (5.3 ± 1.2 mm), surrounded by trichomes and smaller spines (Fig 2C, 2D and 2E).

**Fig 3:**
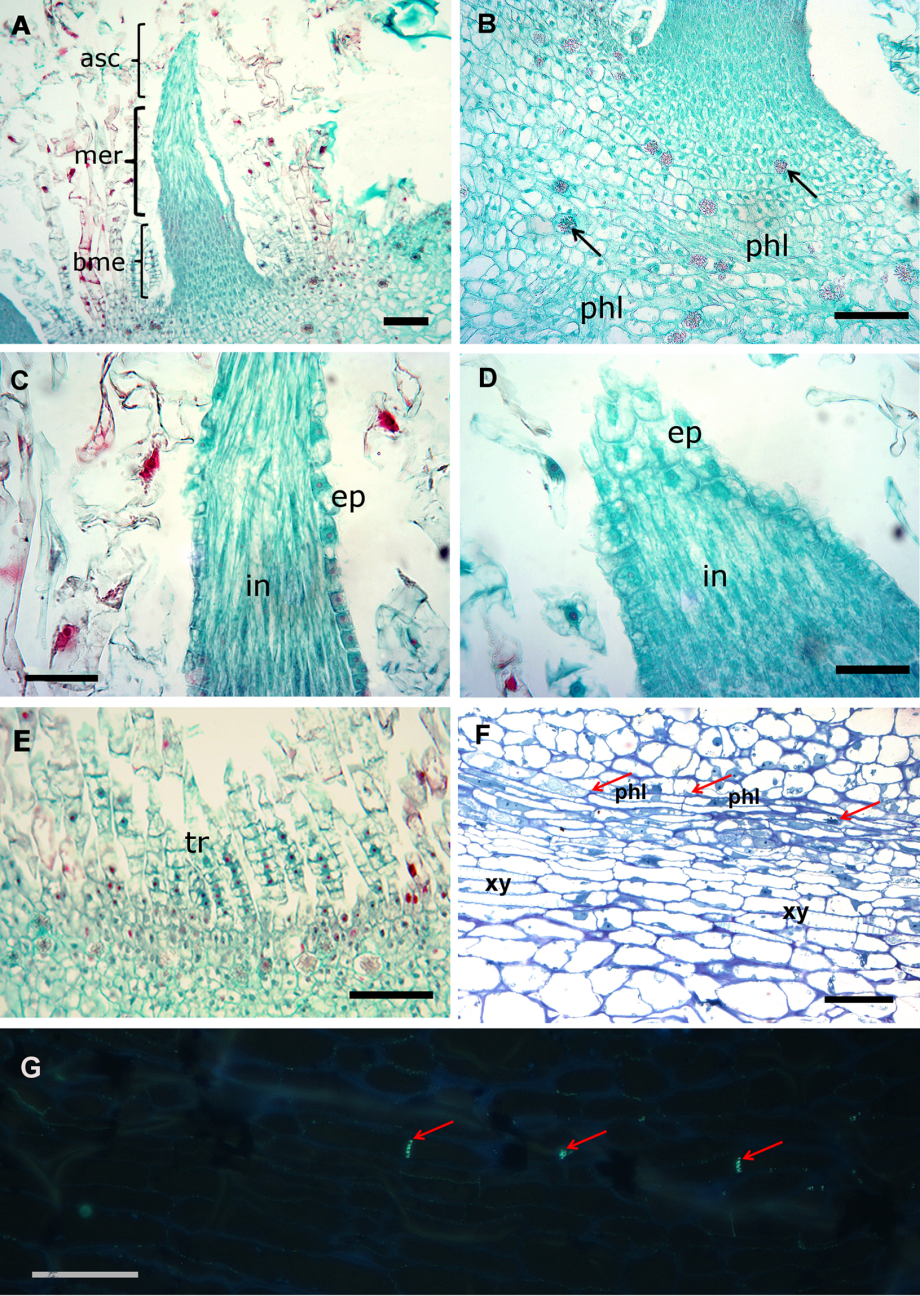
Anatomy of the secreting spine (EFN) of *Opuntia robusta*. (A) Whole spine showing three structural regions (asc, mer, bme); (B) Basal meristem (bme) and areolar tissue beneath the spine base: black arrows indicate crystals; (C) Middle elongation region (mer); (D) apical secreting cone (asc); (E) Trichomes surrounding the EFN; (F) Xylem (xy) and phloem (phl) located beneath the spine base; (G) Aniline blue and epifluorescence of phloem: red arrows indicate fluorescence of callose at the plate of sieve elements (filter excitation BP 365 nm and emission LP 397 nm); in—internal tissue, ep—epidermis, tr—trichomes,. (A), (B), (C)–bar = 100 μm; (D) (E), (F), (G)–bar = 50 μm.

Extrafloral nectaries (EFNs) consist of three distinct regions: 1) a basal meristematic tissue (bme); 2) a middle elongation region (mer); and 3) and apical secretory cone (asc) (Fig 3A). The basal meristem is characterized by a compact tissue of small dividing cells at the spine base. We also observed crystals (druses) in the parenchyma beneath the spine base and some phloem branches (3B).

The middle region is formed by elongated and vacuolated cells described in detail in “Ultrastructure” section; epidermal cells are more globular with conspicuous nucleus, thicker cell walls but not lignified (Fig 3C). The apical secretory cone (asc) has global epidermal cells (Fig 3D), which morphology was better observed with SEM. Light microscopy showed that in young areoles, the young glochids appeared as soft and not lignified multicellular trichomes, composed of 7–12 vacuolated cells with conspicuous nucleus; but no secreting function was detected (Fig 3E).

The EFNs are not internally vascularized, few vascular bundles, including young vessels and phloem elements, leading the young spine, terminate beneath the spine base, and do not penetrate the spine. Semi thin (2 μm) sections of resin, at the base of the spine allowed to identify vessels of xylem (xy) and sieve elements of phloem (phl) (Fig 3F). Sieve elements are characterized by the presence of callose at the sieve plate. Therefore, we confirmed the presence of this type of tissue, and identified it with aniline blue, a callose-specific fluorescent staining (Fig 3G).

**Fig 4:**
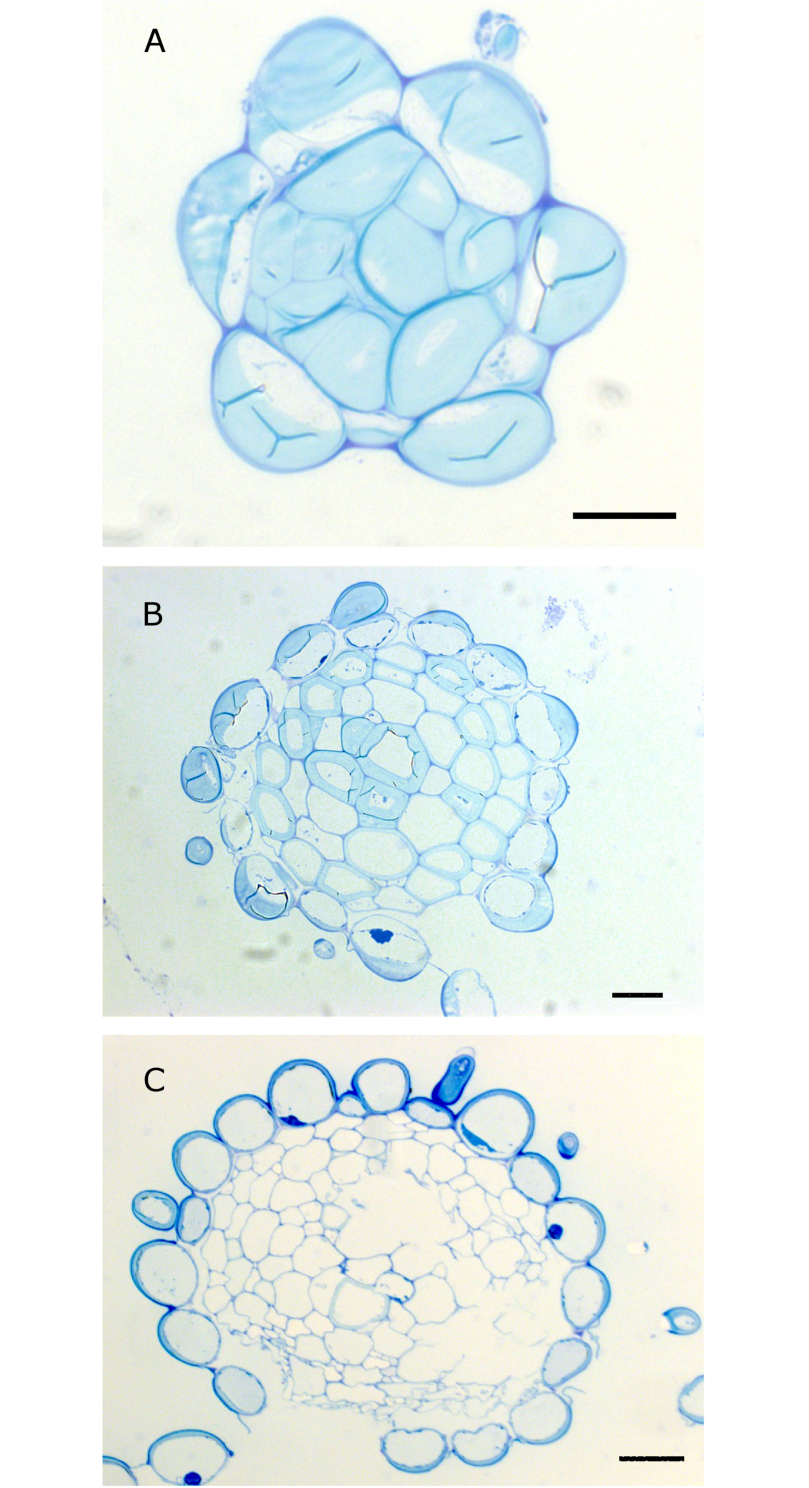
Lignification of the apical cone. (A) Transversal resin section (1 μm) of the apical region (ap) (B) Transversal section of the subapical (sap) region; (C) Transversal region of the basal region of the cone (bac). Observe the thick cell walls of the apex (ap) and some central cells of the subapical region. (A), (B) and (C) bar = 20 μm; (D) bar = 100 μm.

The EFNs lignified acropetally after its secreting function had ended (Fig 4). The apex (ap) of the cone is acute, consisting of small, flattened, elongated cells which are the first to lignify (Fig 4A). Underneath the subapical (sap) part some central cells appeared with thick walls in cross section (Fig 4B) in contrast with the basal region (bca) which is less lignified (Fig 4C).

**Fig 5:**
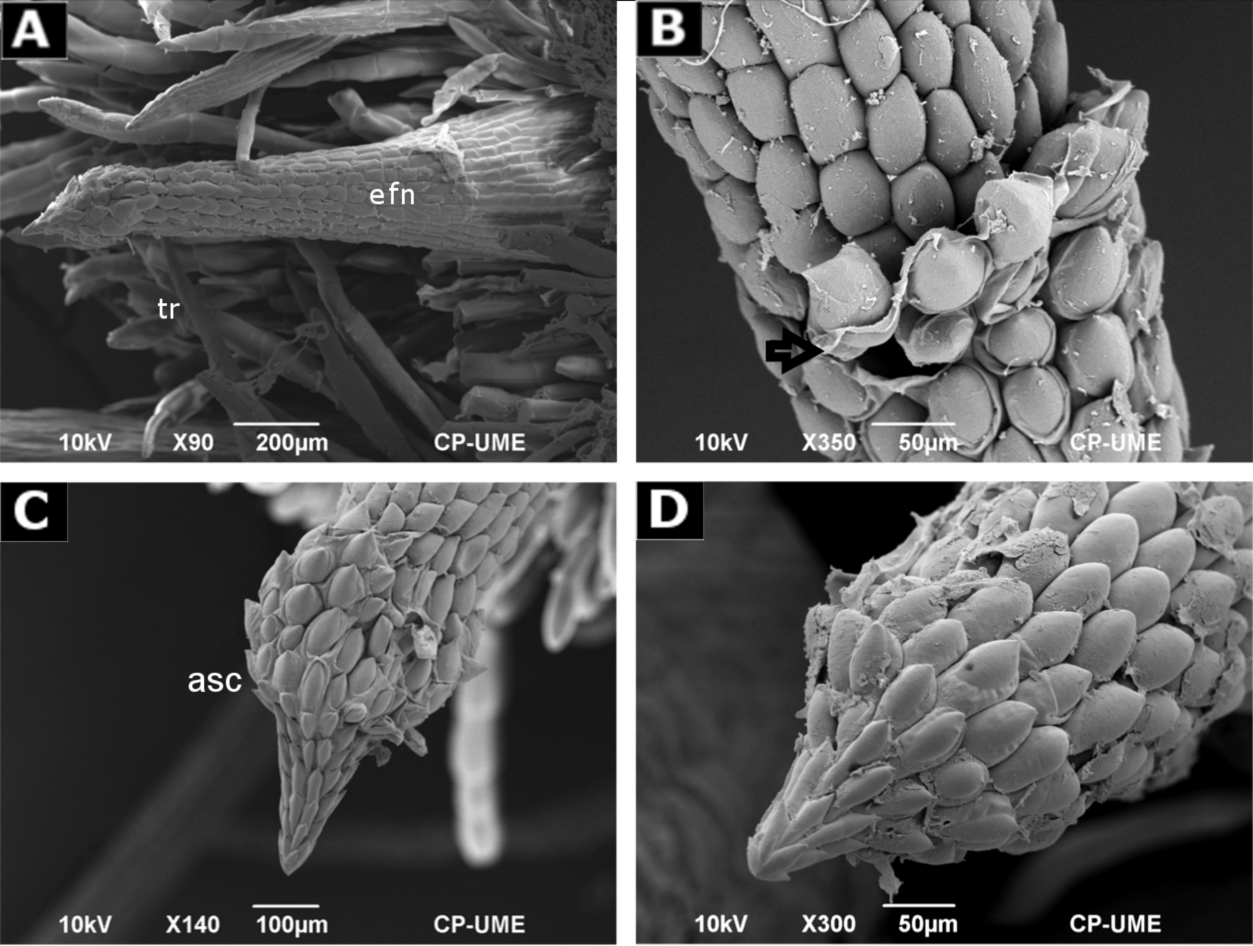
Scanning Electron Micrograph of extrafloral nectary spine. (A) Whole modified spine; (B) Transition region between middle and apical secreting cone showing intercellular spaces and a broken cell; (C) Apical secreting cone; (D) Detail of the apex. asc—apical secreting cone; efn—extrafloral nectary; tr—trichome. (A)—(C)—hermaphrodite, (D)–feminine.

External morphology of the EFNs was better observed with SEM. The apical secretory cone (asc) showed globular and imbricated epidermal cells, with extended cell walls that fold back, like sacs covered by a thin cuticle, resembling an artichoke-like shape (Fig 5).

### Ultrastructure

**Fig 6:**
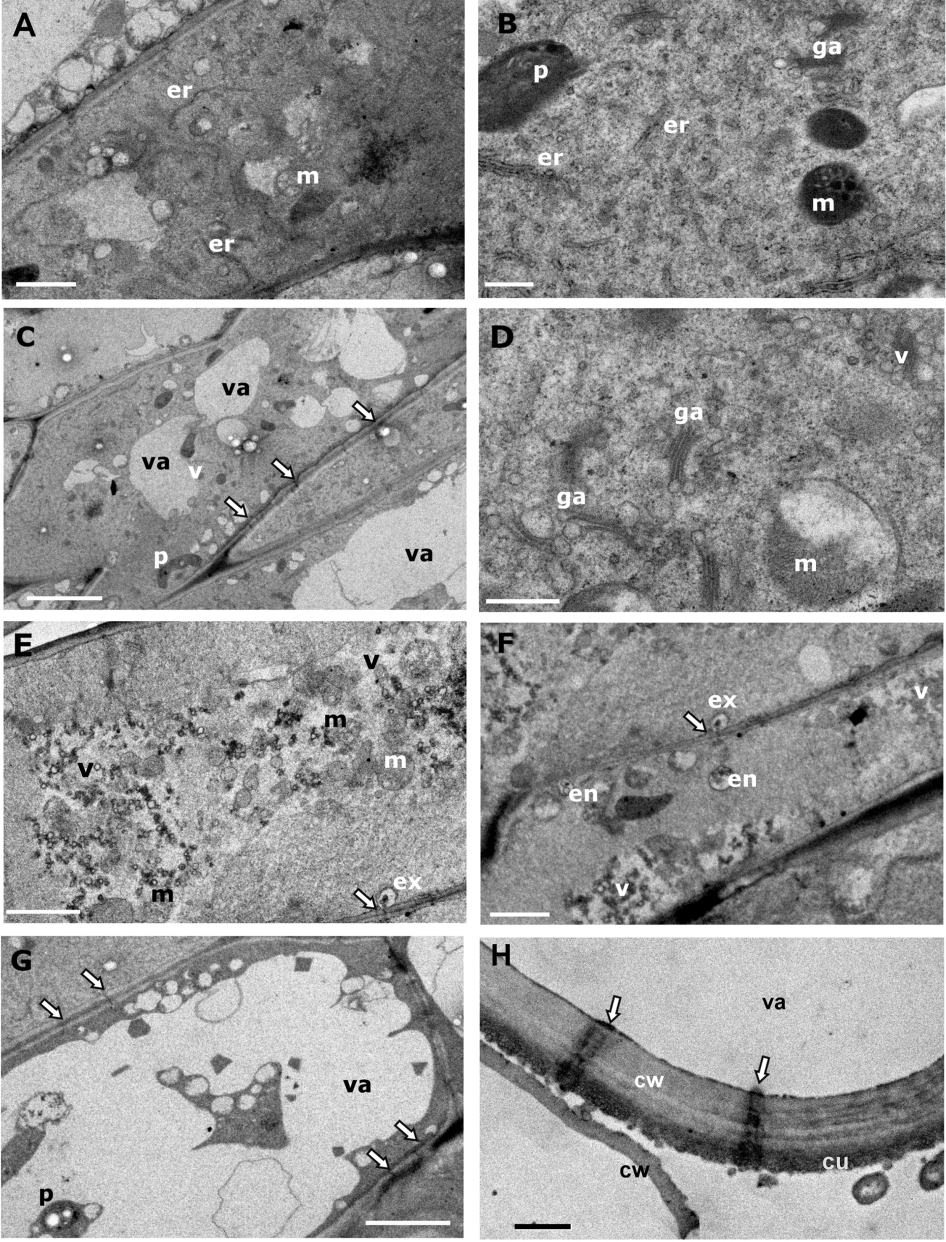
Ultrastructure of extrafloral nectary spine. (A) Basal cell; (B) Detail of a basal cell showing active endoplasmic reticulum and mitochondria; (C) Middle elongated cells showing some vacuoles and numerous pits (arrows); (D) Detail of middle cells showing abundant Golgi apparatus forming vesicles; (E) Internal cell close to the apical secreting cone showing abundant vesicles and mitochondria; (F) Sub epidermal cell, showing vesicle transport between cells by exocytosis and endocytosis; (G) Epidermal cell below the apical secreting cone; with large vacuoles and numerous pits; (H) Epidermal cell of apical secreting cone (nectar reservoirs) showing cuticle and conspicuous pits (arrows), and external granulose material (nectar). cu—cuticle; cw—cell wall; ex—exocytosis; en—endocytosis; ga—Golgi apparatus; er—endoplasmic reticulum; m—mitochondria; va—vacuole; arrows indicate pits at the cell wall. (A) and (E)–bar = 2.0 μm; (B) and (D)–bar = 0.5 μm; (C) and (G)–bar = 5.0 μm; (H) bar = 1 μm.

Cellular structure of the spine provides evidence of its secreting function. The basal region showed cells with dense cytoplasm, thin cell walls, small vacuoles, numerous mitochondria, abundant rough endoplasmic reticulum; and evidence of its active protein synthesis, typical of a meristem (Fig 6A). Proplastids and plastids with few thylakoids are present at the basal meristem and medium region (Fig 6B). At the middle region, internal cells have large vacuoles with some plastids, numerous mitochondria and Golgi apparatus (ga) but less endoplasmic reticulum (re) (Fig 6C). The most conspicuous characteristic of this tissue is the presence of abundant pits and plasmodesmata and active Golgi apparatus with swollen edges forming vesicles (Fig 6C and 6D). Internal cells of the cone share some characteristics with the middle region (mitochondria and plastids), but some cells of the secreting cone are full of vesicles showing a high activity of vesicle transport by exocytosis and endocytosis, where vesicles are invaginated by the cell membrane and excreted to the neighboring cell through the plasmodesmata of pits, then received by plasma membrane invagination of the neighbor cell, all of which suggests the movement of nectar or prenectar to the epidermal cells (Fig 6E through 6G). There was structural evidence of vesicles movement from subepidermal to epidermal cells (Fig 6G). TEM revealed epidermal cells with thick cell walls, large vacuoles that function as nectar reservoir and numerous pits (Fig 6G and 6H). The presence of conspicuous pits and granular substance at the external cell wall suggests the secretion of nectar through pits, due to the osmotic pressure achieved in the epidermal cells.

## Discussion

At the beginning of its development, cladode sprout of *O*. *robusta* is round and approximately symmetric; later, it turns flatter, longer, wider and more asymmetric [104]. Ants preferred narrower sprouts that correspond to their earlier stage of development, where young secreting spines were active. The only size trait associated with the humidity was sprout thickness, however, it increased only slightly with the increase of air humidity, and the dispersal of the data was too large to significantly explain this relationship when other variables were controlled. Also, ants’ number on sprouts showed an increasing tendency, with the increase of sprout thickness together with air humidity. Even when abiotic variables were correlated, the effect of each of them showed different strengths and significances when studied together in the PMRM. For example, the simple relationship between air humidity and temperature explained 97% of the variability, but the association of the ants’ average number on the cladode sprout with the former variable was to some extent independent from the association with the latter variable, since whereas the effect of humidity was close to be significant (*P* = 0.065), the effect of temperature was clearly non-significant. Also, sprout width and length were associated independently with the ants’ number, provided that both variables were strongly correlated, but only the former had a significant effect when studied together with the latter in the PMRM: sprout length was associated with ants’ number only at the edge of significance (*P* = 0.07). This could be an effect of some unknown correlation between the level of spine development (and thus, intensity of secretion of extrafloral nectar) and cladode width and length, or of unknown factors that explained 12% of the model. More intense ant foraging on smaller cladode sprouts is concordant with ODH [46] that has been invoked to explain why younger leaves are better defended than older ones [105, 106]. In the case of *O*. *robusta*, cladodes play the same physiological role as leaves and, following ODH, cladode sprouts should be better defended than adult cladodes. Even when classical ODH refers to defensive substances, similarly to their production, the production of nectar requires energy because in most cases it contains sugars [43, 107, 108] or sugars and other organic compounds [3–7], and its indirect deterrent effect on herbivores improves fitness by preventing the loss of photosynthetic biomass. One of the possible explanations for very few observations of ant aggressive behavior toward true herbivores is the lack of herbivore species against which EFNs had evolved in the past. This may be possible because of several environmental transformations that fragmented the scrubland in this zone during the last years (2007–2016) and that we could observe during our study. Some ant species produce pheromones that may be perceived by herbivores as long-lasting warning signals. A positive correlation between pheromone persistency of the *Oecophylla* F. Smith, 1860 ant and plant protection efficiency was found [109, 110]. Future studies should be carried out to confirm if the observed lack of ants’ attacks on herbivores is attributable to such phenomenon. We suggest a possibility that EFNs on cladodes sprouts in this plant have evolved as an indirect response to herbivory rather than to distract ants from pollinators as proposed by distraction hypothesis, or attraction of ant nests as proposed by nutrition hypothesis, because of four reasons: 1) these nectaries are present also on cladode sprouts of plants that are not blossoming and generally, the distraction of ants from attacking pollinators occurs when EFNs are placed either close or on the external section of a flower [35, 36]; additionally, in our study zone a clear trade-off occurs between the production of flower and cladode sprouts: they are produced very rarely on the same parental cladode [65, 104]; 2) We also discovered EFNs on the external part of the flowers and young fruits of *O*. *robusta*: if ants’ distraction from pollinators occurs, it is more likely performed by these EFNs; 3) The variable that better explained the presence of ants was sprouts of small width which, in turn, contain immature spines with secreting cones that produce nectar. 4) Ant nest attraction hypothesis (or, nutrition hypothesis) is very unlikely as a main selective force because, to occur, it does not need the existence of ants’ aggressive behavior; however, it could have originated as a side effect of the other two (defense and distraction).

EFNs in *O*. *robusta* differ from EFNs of others plant species. Secretion occurs in modified young spines located behind the persistent leaf of areoles. Nectary glands in the Cactaceae species such as in *A*. *scheeri* are produced in the shoot apical meristem next to areoles; gland primordia expand and differentiate into a stalk region and a glandular region, highly vascularized [16]. In contrast, in *O*. *robusta*, EFNs are non-vascularized secreting spines, where morphology was modified externally and internally to perform nectar production, transport, accumulation, and secretion. The cellular structure of the cone suggests its secretory function; nectar is produced by internal and subepidermal cells and transported to the epidermal cells of the apical cone that function as nectar reservoir. Later, the nectar can be released through the pits or by ants biting the apical cone.

A secretory tissue may consist of cells involved in the elimination of the secreted substance directly into extracellular spaces or to auxiliary cells [111]. In *O*. *robusta* the production of nectar and its secretion is compartmentalized; internal cells of the modified spine show a typical ultrastructure of secreting cells, characterized by numerous Golgi apparatus, vesicles, mitochondria, and endoplasmatic reticulum. We observed many vesicles in contact with the plasmalemma, similar to the nectary cells of banana flowers [112]. Our observation in *O*. *robusta* suggests a nectar transport described as eccrine secretion by Fahn & Benouaiche [112] and by Fahn [113]; besides, we showed evidence of granulocrine transport of vesicles between cells, where endocytosis and exocytosis occur. The presence of numerous pits and plasmodesmata in *O*. *robusta* is a characteristic described for EFNs of *Vicia faba* L. [114], cotton *Gossypium hirsutum* L. [115], and the floral nectaries of *Lonicera japonica* Thunb. [116], *Thunbergia laurifolia* Lindl. [117], and *Peganum harmala* L. [118].

In *O*. *stricta*, EFNs were described as flat-topped glands located on the areoles of developing tissue in young cladodes, beneath thorns; they are vascularized and consist of palisade secretory parenchyma, covered by a thick cuticle, which breaks down to release nectar and these glands are raised on a densely clothed base with soft non-secretory trichomes [33]. The morphology and anatomy observed in the EFNs of *O*. *robusta* differ from the types of EFNs described in *O*. *stricta* and *A*. *scheeri* members of the Cactaceae family, and from other species described by Zimmermann [2] and Fahn [111]. Release of nectar from the nectary varies among species: in *Lonicera* L. the one-celled secretory hair is accumulated below the cuticle and has no pores [119]; in *Vinca* sp. L. the nectar is secreted through the walls without cuticle, into intercellular spaces [120]; and the cuticle breaks in *O*. *stricta* [33]. In *O*. *robusta* we found pits on the external epidermis walls of the apical cone, similar to those reported in two horticultural forms of *Abutilon* [121].

In *L*. *japonica* [116] and two species of *Vinca*, *and Citrus sinensis* Osbeck cv. Valencia [120], secretion of nectar is associated with a pronounced increase of ER, Golgi apparatus; therefore, both organelles would participate in the process. In *O*. *robusta* only Golgi apparatus and vesicles were abundant in the internal tissue of the spine, suggesting its role in the production and transportation of the nectar [122].

Lloyd & Ridgway [123] stated that nectar in *Opuntia* is released after rupture of epidermal cell wall. Our TEM observations on *O*. *robusta* suggested that nectar may be released through pits of the epidermal cell wall of the secreting cone, but in the field we observed that ants manipulated the cone and extracted the nectar. At maturity, secretory spines (EFNs) lignify and they stop secreting nectar.

Gibson & Nobel [124] suggested that the absence of vascular traces inside spines is explained by the faster lignification of the spine. The EFNs of *O*. *robusta* has an acropetal development; lignification starts at the very tip and continue toward the base. The lack of vascular tissue in the secretory spines of *O*. *robusta* seems to be more related to its juvenile condition and the presence of a basal meristem. Although the secreting activity is temporary, it is valuable for protecting the young cladodes against herbivores.

In our study, the presence of plastids in the secreting spine indicates photosynthetic activity associated with nectar production [125, 126].

In several plants, calcium oxalate (druses) is associated with calcareous soils or Ca^2+^ excess in the tissue. Giaquinta [127] showed that Ca^2+^ is involved in sucrose transport in plant tissue, but in *O*. *robusta*, druses are very common crystals in the parenchyma tissue.

Trichomes of *O*. *robusta* and trichomes of *Abutilon* sp. Mill. are similar in their origin from successive periclinal divisions of one epidermal cell and their vacuoles placed laterally to the large nucleus [128, 129].

The ultrastructure of the EFNs of *O*. *robusta* indicates that pre-nectar is transported through pits and plasmodesmata by exocytosis and endocytosis mechanisms along the spine, it is stored on the epidermal cells of the apical cone, and nectar secreted through pits or released due to ant’s manipulation to extract the nectar is also a possibility.

The comparison of EFNs of *O*. *robusta* and *O*. *stricta* suggests that the hypothesis of Diaz-Castelazo et al. [33] should be revised: the EFNs *O*. *stricta* and *O*. *robusta* show similar among-organs distribution, whereas their vascularization differs; in *O*. *robusta* EFNs are vascularized, but not in *O*. *stricta*. Therefore, the presence or the type of EFNs is not a good taxonomic trait in this genus. This study contributes to the knowledge of the morphology, anatomy, structure and possible defensive role of EFNs in *O*. *robusta*, a species from the genus that encompasses several species producing EFNs, but structurally different. Taking into account these findings and of other studies [33, 72], a modification can be added to the hypothesis of Diaz-Castelazo et al. [33]: circumscribed multicellular EFNs can be either vascularized on non-vascularized, including secretory spines.

### Conclusions

EFNs in *O*. *robusta* and in several *Opuntia* species are modified young spines placed on areoles. Diaz-Castelazo’s hypothesis states that more complex EFNs structure correlates with their lower among-organs dispersion, comparing to less complex EFNs. However, non-vascularized (less complex) secretory young spines in *O*. *robusta* are placed in the same organs (young cladode in flower sprouts) as vascularized (more complex) young spines in *O*. *stricta*. This comparison shows that the presence or the absence of vascularization, and thus, the level of complexity of EFNs, may not be as strongly correlated with the pattern of their distribution among different organs as stated by Diaz-Castelazo’s hypothesis. We suggest that the level of complexity of EFNs is an effect of an independent evolution of these structures in closely related species: natural selection shapes the tissues’ response to biotic conditions rather than the position of EFNs on a given organ. Our study is the first description of the anatomy and ultrastructure of extrafloral nectaries in *Opuntia robusta* (Cactaceae).

## Supporting information

S1 Fig The relationship between relative air humidity and air temperature (A) or sampling time (B), and between air temperature and sampling time (C). (PDF)

S2 Fig The relationship between cladode length (A), width (B), or thickness (C) and air temperature, as well as between cladode length (D), width (E), or thickness (F) and relative air humidity. (PDF)

S1 Table The number of ants observed on sprouts with a given size (length, width, thickness), together with air temperature and humidity recorded at the moment of sampling. (XLSX)

S1 Video Ants’ activity on cladode sprouts. Ants searched mainly the apical secretory cones of young spines in areoles. We observed the secretion of transparent liquid, which attracted and fed ants.
(MP4)
