# Supplementary material for: First description of extrafloral nectaries in Opuntia robusta (Cactaceae): Anatomy and ultrastructure
Source: PLoS One. 2018 Jul 17;13(7):e0200422. doi: 10.1371/journal.pone.0200422 (PMC6049920; doi:10.1371/journal.pone.0200422)
Supplement: S1 Fig — (PDF) [file pone.0200422.s001.pdf]

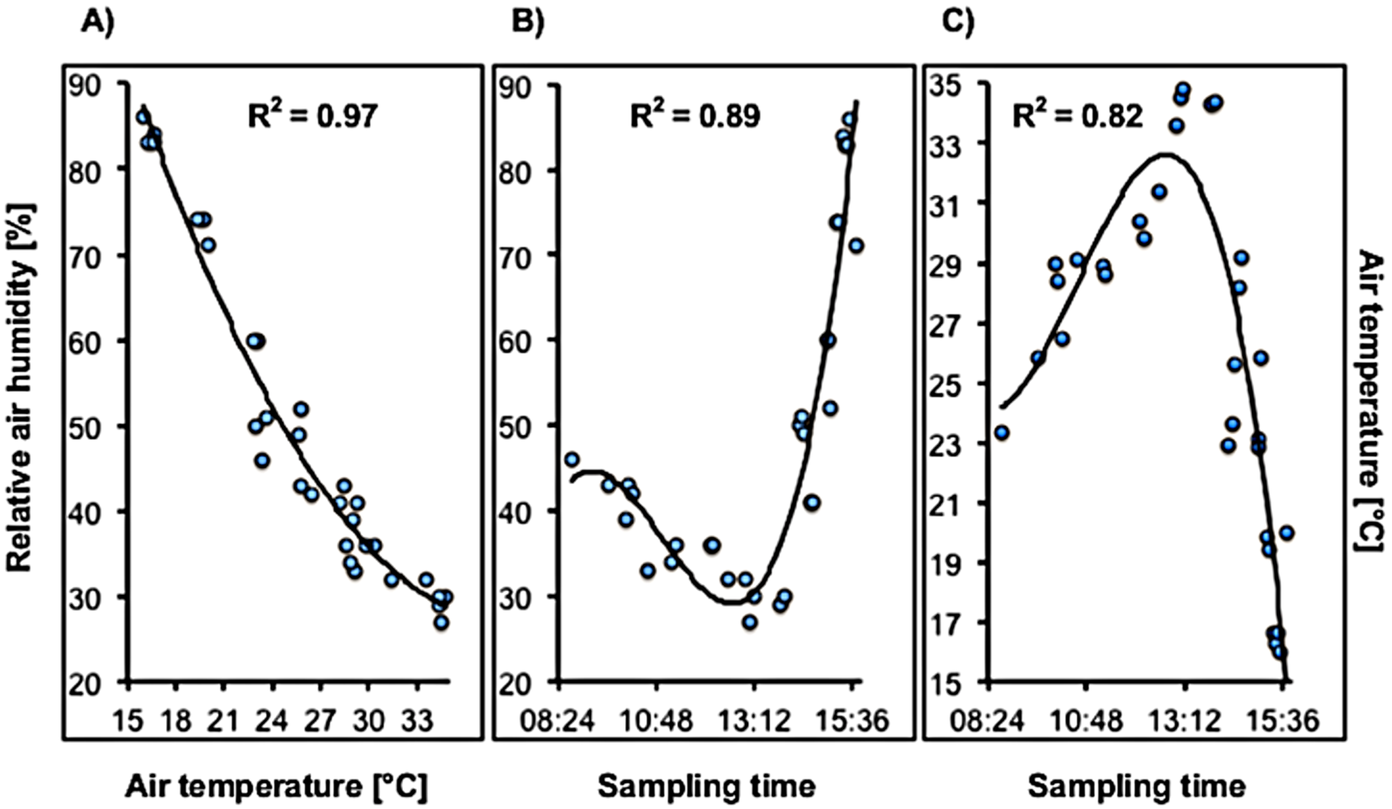
A)
B)
C)
R² = 0.97
R² = 0.89
R² = 0.82
Relative air humidity [%]
Air temperature [°C]
Air temperature [°C]
Sampling time
Sampling time
90
80
70
60
50
40
30
20
15 18 21 24 27 30 33
08:24 10:48 13:12 15:36
35
33
31
29
27
25
23
21
19
17
15

S3 Figure. The relationship between relative air humidity and air temperature (A) or sampling time (B), and between air temperature and sampling time (C). These relationships were described by the fallowing least-square equations and their adjustment by the fallowing coefficients of determination (R^2): A) $y = 0.12x^2 - 9.14x + 203.11$, $R^2 = 0.97$; B) $y = 0.76x^3 - 5.09x^2 + 4.53x + 43.56$, $R^2 = 0.89$; C) $y = - 0.21x^3 + 1.21x^2 + 0.71x + 24.17$, $R^2 = 0.82$. We transformed the variable "time" in the fallowing way: $h' = h \times 24 - \min(h) \times 24$, where h' – transformed time, h – time in original units, $\min(h)$ – the earliest sampling hour. For all relationships, all the effects (linear, quadratic, and cubic) were significant ($P < 0.0001$).
