# Supplementary material for: First description of extrafloral nectaries in Opuntia robusta (Cactaceae): Anatomy and ultrastructure
Source: PLoS One. 2018 Jul 17;13(7):e0200422. doi: 10.1371/journal.pone.0200422 (PMC6049920; doi:10.1371/journal.pone.0200422)
Supplement: S2 Fig — (PDF) [file pone.0200422.s002.pdf]

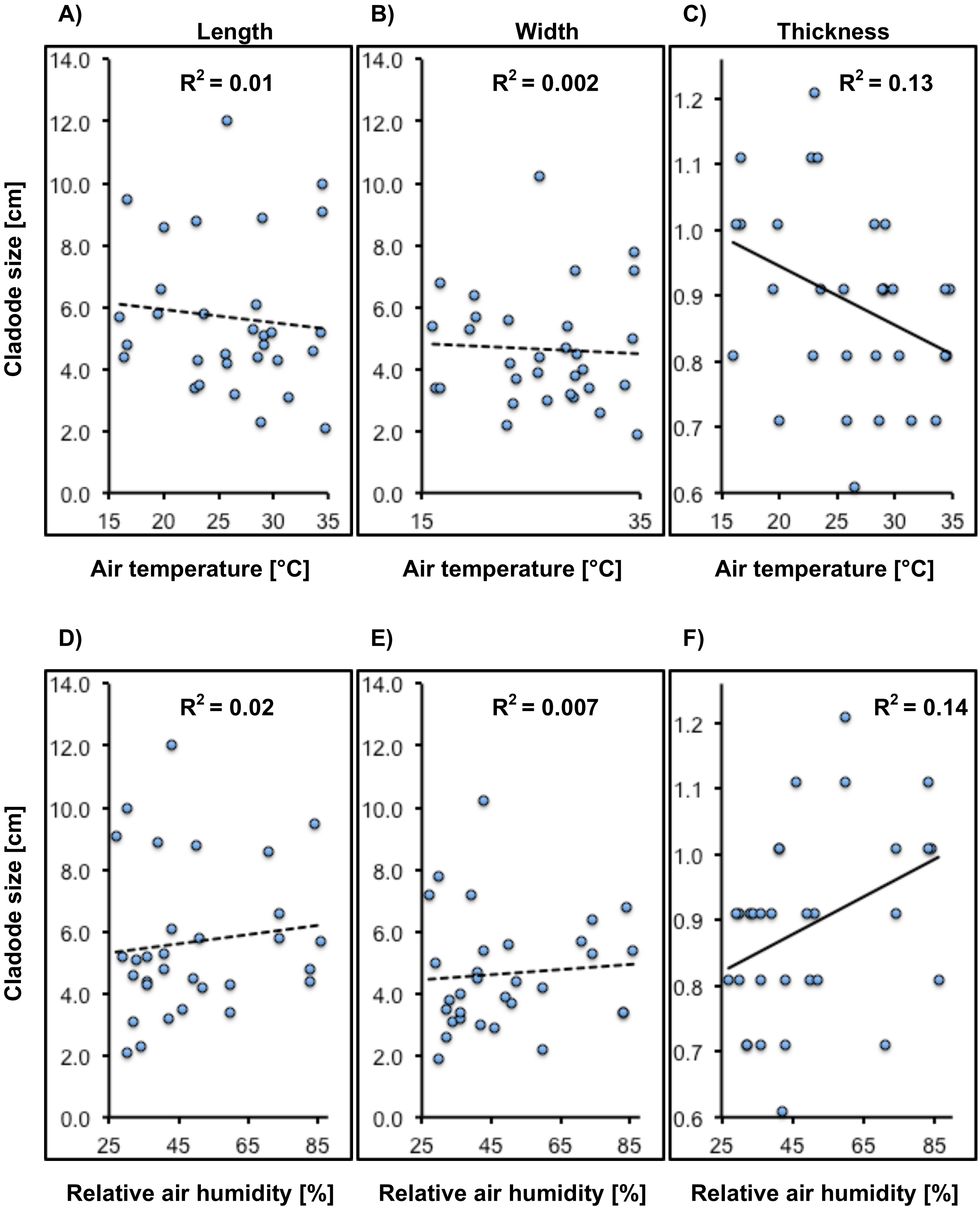

A)
Length
B)
Width
C)
Thickness
R2 = 0.01
R2 = 0.002
R2 = 0.13
Cladode size [cm]
Air temperature [°C]
Air temperature [°C]
Air temperature [°C]
D)
E)
F)
R2 = 0.02
R2 = 0.007
R2 = 0.14
Cladode size [cm]
Relative air humidity [%]
Relative air humidity [%]
Relative air humidity [%]

S4 Figure. The relationship between cladode length (A), width (B), or thickness (C) and air temperature, as well as between cladode length (D), width (E), or thickness (F) and relative air humidity. These relationships were described by the fallowing least-square equations, and their adjustment by the fallowing coefficients of determination (R^2): A) y = -0.04x + 6.8, R^2 = 0.01, *P* = 0.6; B) y = -0.02x + 5.07, R^2 = 0.003, *P* = 0.8; C) y = 7e-05x2 - 0.013x + 1.16, R^2 = 0.13, ***P* = 0.05**; D) y = 0.015x + 4.9, R^2 = 0.013, *P* = 0.5; E) y = 0.008x + 4.2, R^2 = 0.007, *P* = 0.7; F) y = 0.003x + 0.7, R^2 = 0.14, ***P* = 0.04**. Regression equations for the significant relationships are depicted with continuous line. Significant relationships are marked with bold.
